# Microstructural and Compositional Features of the Fibrous and Hyaline Cartilage on the Medial Tibial Plateau Imply a Unique Role for the Hopping Locomotion of Kangaroo

**DOI:** 10.1371/journal.pone.0074303

**Published:** 2013-09-18

**Authors:** Bo He, Jian Ping Wu, Jiake Xu, Robert E. Day, Thomas Brett Kirk

**Affiliations:** 1 School of Pathology and Laboratory Medicine, The University of Western Australia, Crawley, Australia; 2 Department of Mechanical Engineering, Curtin University, Bentley, Australia; 3 Department of Medical Engineering & Physics, Royal Perth Hospital, Perth, Australia; University of Minho, Portugal

## Abstract

Hopping provides efficient and energy saving locomotion for kangaroos, but it results in great forces in the knee joints. A previous study has suggested that a unique fibrous cartilage in the central region of the tibial cartilage could serve to decrease the peak stresses generated within kangaroo tibiofemoral joints. However, the influences of the microstructure, composition and mechanical properties of the central fibrous and peripheral hyaline cartilage on the function of the knee joints are still to be defined. The present study showed that the fibrous cartilage was thicker and had a lower chondrocyte density than the hyaline cartilage. Despite having a higher PG content in the middle and deep zones, the fibrous cartilage had an inferior compressive strength compared to the peripheral hyaline cartilage. The fibrous cartilage had a complex three dimensional collagen meshwork with collagen bundles parallel to the surface in the superficial zone, and with collagen bundles both parallel and perpendicular to the surface in the middle and deep zones. The collagen in the hyaline cartilage displayed a typical Benninghoff structure, with collagen fibres parallel to the surface in the superficial zone and collagen fibres perpendicular to the surface in the deep zone. Elastin fibres were found throughout the entire tissue depth of the fibrous cartilage and displayed a similar alignment to the adjacent collagen bundles. In comparison, the elastin fibres in the hyaline cartilage were confined within the superficial zone. This study examined for the first time the fibrillary structure, PG content and compressive properties of the central fibrous cartilage pad and peripheral hyaline cartilage within the kangaroo medial tibial plateau. It provided insights into the microstructure and composition of the fibrous and peripheral hyaline cartilage in relation to the unique mechanical properties of the tissues to provide for the normal activities of kangaroos.

## Introduction

The locomotory characteristics of kangaroos are unusual, with gait parameters differing from those of humans and quadrupedal running mammals. A large kangaroo hops at an average speed of 40 km h^−1^ for several kilometres and may reach a speed of 50–65 km h^−1^ in short bursts [1]. During high speed hopping, kangaroos lift off and land on the ground simultaneously with both feet. This unusual form of locomotion results in high ground reaction forces and high loads in the kangaroo knee joints [2].

During normal walking, forces generated in human knee joints are about 2 to 4 times of the body weight [3]. The ground reaction force of the kangaroo during hopping is several times higher than that of human’s walking and running activities [2], [4]. Furthermore, hopping kangaroos have a higher stride frequency when progressing at the same velocity [2]. Thus, greater and more frequent dynamic stresses are expected in the knee joints of kangaroos. A previous study has reported that kangaroo tibia possess an unusual fibrous cartilage pad in the centre, which was surrounded by hyaline cartilage [2]. Such a unique tibial anatomy has been suggested to be crucial for the kangaroo knee to adapt to the prevailing high loads and speed requirements during the locomotion [2]. The fibrous pad has been reported to be softer and more compressible than the surrounding hyaline cartilage. This enabled the pad to deform more easily to enlarge the articular contact area, thus decreasing the high frequent peak stresses generated in the knee. It is well recognised that the fibril structure and PG composition gives rise to the mechanical properties of articular cartilage, which play a crucial role in the normal function of knee joints. Therefore, detailed study of the compositional, microstructural and mechanical characteristics of the fibrous cartilage and the peripheral hyaline cartilage will offer greater understanding of the unique function of the fibrous pad and kangaroo tibial plateau in the animal’s locomotion The study examined the structural arrangement of collagen and elastin fibres, PG content and mechanical properties of the fibrous and hyaline cartilage in the medial tibia of kangaroo knees.

## Materials and Methods

### Specimen Preparation

**Figure 1:**
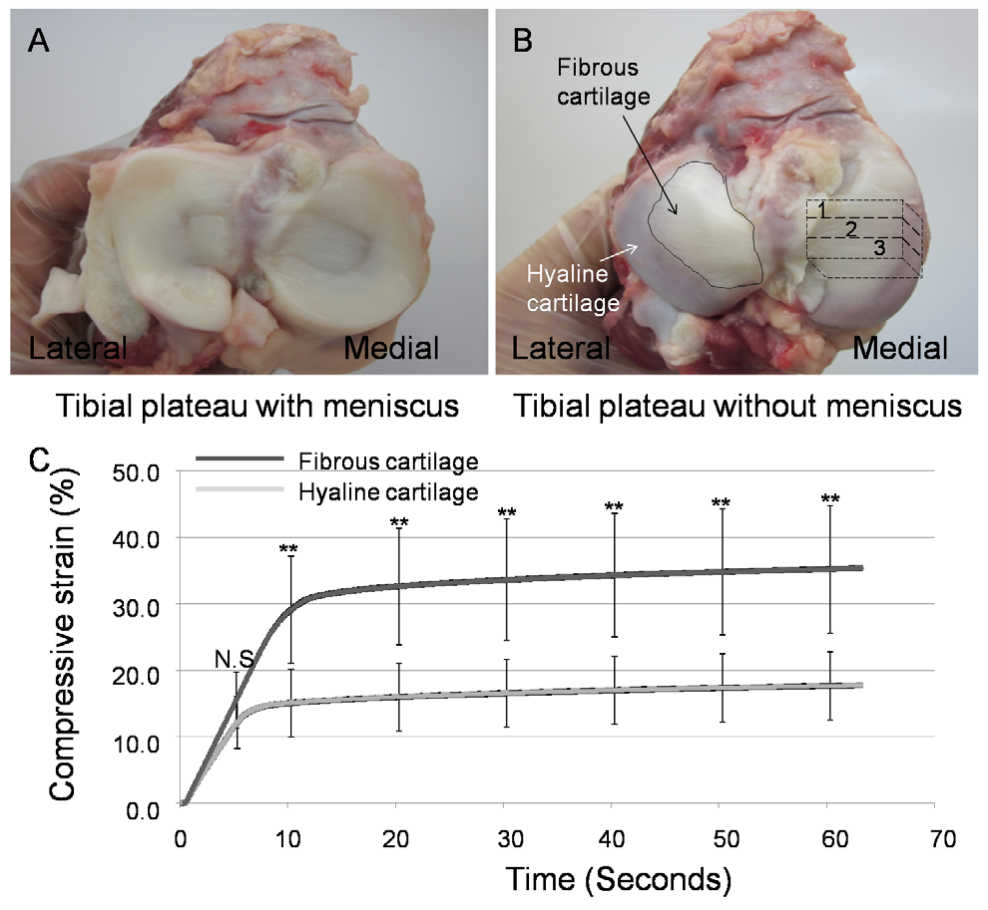
The anatomy of the articular surface of kangaroo tibial plateau, and the creep behaviour of fibrous cartilage and hyaline cartilage. Menisci cover approximately 70% of the medial tibial plateau and 80% the lateral tibial plateau (A). The fibrous cartilage is located in the central area and accounts for approximate 55% of the medial and 50% of the lateral tibial plateau surfaces (B). The middle area of tibial plateau was marked divided into three parts (#1, 2 and 3) and the region #2 was subjected to indentation tests. Higher compressive strain in the fibrous cartilage shown from 10 seconds to 60 seconds of the test indicated that the fibrous cartilage was more readily deformed than hyaline cartilage (**P<0.0001) (C). N.S = not significant.

Five whole knee joints were collected from five male western gray kangaroos (*Macropus fuliginosus*), each about five years old, from a local butcher (King River International Company, Perth, Australia) and the tibial plateaus were dissected out (Fig. 1A & 1B). The cartilage surfaces were visually inspected for the absence of osteoarthritis (OA). For each knee a specimen block that included both the fibrous and peripheral cartilage down to the subchondral bone was cut from the medial tibial plateau using a hacksaw. The block was then marked into three sections (#1, #2 and #3, Fig. 1B).

The middle section (#2, Fig. 1B) was used for mechanical testing, after which the block was dissected into three parts along the marked lines. The first section (#1, Fig. 1B) was used for traditional histology; the third section (#3, Fig. 1B) was used for imaging of the collagen and elastin by multiphoton microscopy. For this study the third section (#3, Fig. 1B) was further cut longitudinally into two equal parts. One part was used for imaging in the longitudinal view and the other part was used for imaging in the transverse view.

### Ethics Statement

This study was carried out in strict accordance with the recommendations from the Animal Ethics Committee of the University of Western Australia. The Animal Ethics Committee of the University of Western Australia approved the present protocols. Permission was also obtained from the local butcher (King River International Company, Perth, Australia) to use the kangaroo joints and the tibial plateaus for research purpose.

### Pin Indentation Creep Test

There are several ways to perform creep measurements in articular cartilage [5]–[7]. Pin indentation creep testing has the advantage of being able to make measurements at multiple locations within a single tissue sample and was used in this study to compare the mechanical behaviour of the central fibrous and peripheral hyaline cartilage on the kangaroo tibia in relation to their microstructure and composition.

A universal testing machine (Instron 5848 microtester; Instron, Norwood, MA, USA) equipped with a 100 N Load cell and 2 mm diameter indenter was used to test the creep response of the cartilage. For each of the tibial plateau blocks, 5 locations within the fibrous cartilage and 3 locations within the hyaline cartilage were tested. A pilot study showed that the fibrous cartilage collapsed completely during pin indentation with loads much above 0.3 N. In addition, the load-deflection curve for the fibrous cartilage showed an unusual deflection at around 70 seconds of indentation creep. To avoid these effects a load of 0.3 N held constant for 60 seconds was sued for all the tests in this study. Load and compressive strain were recorded continuously at 0.1 second intervals. Comparative strains were determined individually by calculating the mean value of all the strains from 10 to 60 seconds.

### Histological Analysis

After dissection, full thickness cartilage samples (#1, Fig. 1B) were fixed in 10% neutral buffered formalin solution (10% BFS) for 24 h and decalcified in Rapid Decal (HD Scientific Supplies Pty Ltd.) for 5 h. After being washed with phosphate buffed saline (PBS, pH 7.2), the specimens were serially dehydrated in ethanol and cleaned in toluene. The specimens were then embedded in paraffin blocks and cut in to 5-µm serial slices from the articular surface to the subchondral bone using a rotary microtome (Leica RM2255). The slices were stained with Haematoxylin and Eosin (H&E) for morphological assessment, and with Safranin O – fast green for analysis of the proteoglycan (PG) content.

The slices were scanned using a digital slide scanner (Aperio ScanScope System, Aperio Technologies, Vista, CA) at 20×magnification and the scans evaluated with ImageScope (version 11.1.2.760, Aperio Technologies, Vista, CA).

### Multiphoton Confocal Laser Microscopy for Collagen and Elastin

The imaging of collagen in this study was based on the inherent non-centrosymmetric structure of collagen. Fibrillar collagen possesses a tremendous nonlinear susceptibility and can generate extremely bright and robust second harmonic generation (SHG) signals [8], allowing submicron resolution imaging of the collagen structure. As elastin fibres do not exhibit any SHG signal [9], sulforhodamine B (SRB), which stains specifically elastin [10], was used in the present study. SRB powder (Sigma-Aldrich) was dissolved in 0.9% saline water and 1 mg/ml SRB solution was used for staining of kangaroo cartilage for 1 min. After thorough washing in PBS, cartilage samples were immersed in PBS to maintain tissue hydration and mounted between a coverslip and a glass slide.

Images of the collagen and elastin were acquired using a multiphoton confocal laser scanning microscope (Leica TCS SP2 acousto-optical beam splitter) (AOBS) equipped with several light sources, including a DPSS laser at 561 nm and a multiphoton laser generated by a Spectra Physics Mai Tai sapphire system which could be tuned from 710 to 990 nm. The Mai Tai laser at 890 nm wavelength was used to excite the SHG and the SHG signal from collagen was collected at 445 nm by a Leica oil S2 condenser. The DPSS laser at 561 nm was used to excite SRB and the corresponding signal from elastin was collected at 565 to 590 nm. Laser power and detector sensitivity were adjusted to provide optimum image quality without excessive dye bleaching or pixel saturation. For noise reduction, 1024×1024 pixel images were obtained using a 1-µm step and frame averaging 4 scans per image.

### Statistical Tests

To compare the differences in compressive strain between the fibrous cartilage and hyaline cartilage, the linear mixed effects model was chosen. This model is designed to analyse data characterized by more than one source of variability and allows samples with potential intraclass correlation to be reliably compared [11], [12]. In the model, compressive strain was set as the dependent variable; cartilage type (fibrous or hyaline) and duration were set as fixed variables; with the animal coded as the random variable. Estimated mean and standard deviation of the compressive strain for the fibrous and hyaline regions were present at 5, 10, 20, 30, 40, 50 and 60 seconds. Significance was chosen at *p*<0.05. All statistical analyses and graphs were produced using Statistical Package for the Social Sciences (SPSS), version 16.0 (SPSS Inc., Chicago, IL, USA).

## Results

### Anatomy of Tibial Plateau

Meniscus covers approximately 70% of the medial tibial plateau and 80% the lateral tibial plateau (Fig. 1A). Based on the macromorphology, the tibial plateau of the kangaroo can be categorised into two parts: the fibrous cartilage (black arrow in Fig. 1B) and the hyaline cartilage (white arrow in Fig. 1B). The fibrous cartilage is located in the central area and accounts for approximate 55% of the articular surface of medial tibia and 50% of the articular surface of lateral tibia. This fibrous tissue is partially in direct contact with femoral condyle and partially covered by menisci. The fibrous cartilage surface in the medial tibial plateau is slightly concave in the centre and slightly convex in the lateral tibial plateau (Fig. 1B). The hyaline cartilage is located peripherally on the tibial plateau and fully covered by menisci (Fig. 1B).

### Pin Indentation Creep Test

The pin indentation tests showed that both the fibrous and hyaline cartilage of the kangaroo tibial plateau displayed typical creep behaviours. However, the compressive strain of the fibrous cartilage during 10–60 seconds was more than twice that of the surrounding hyaline cartilage (*p*<0.0001) (Fig. 1C). The mean compressive strain of the hyaline cartilage was about 15%, while the compressive strain of the fibrous cartilage was about 35% (Fig. 1C).

### Histological Characteristics of Tibial Plateau Cartilage

**Figure 2:**
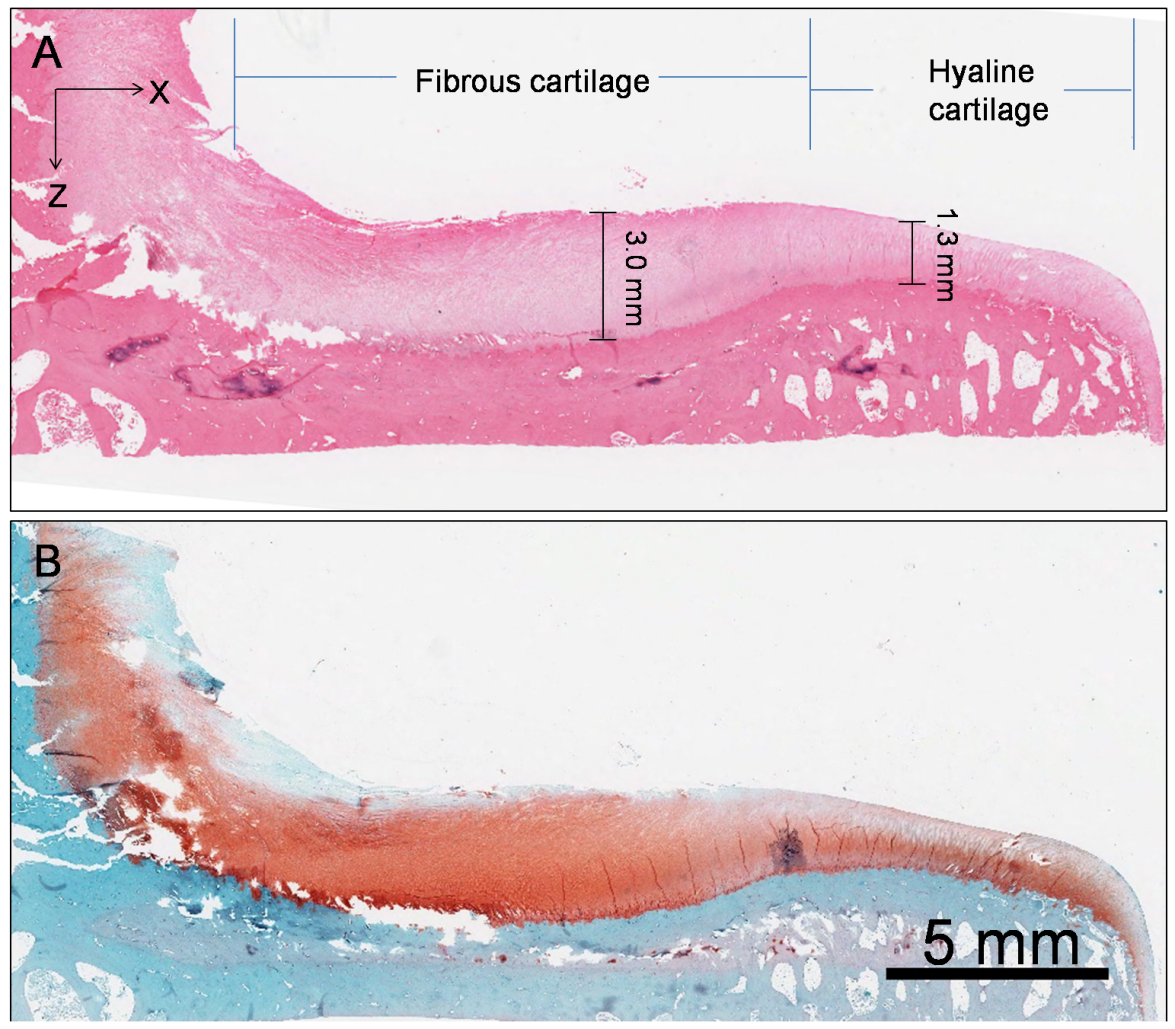
Typical view of histomorphology and PG distribution in the tibial plateau cartilage. The fibrous cartilage was more than two times thicker than the hyaline cartilage (A) and had a higher PG concentration (B).

**Figure 3:**
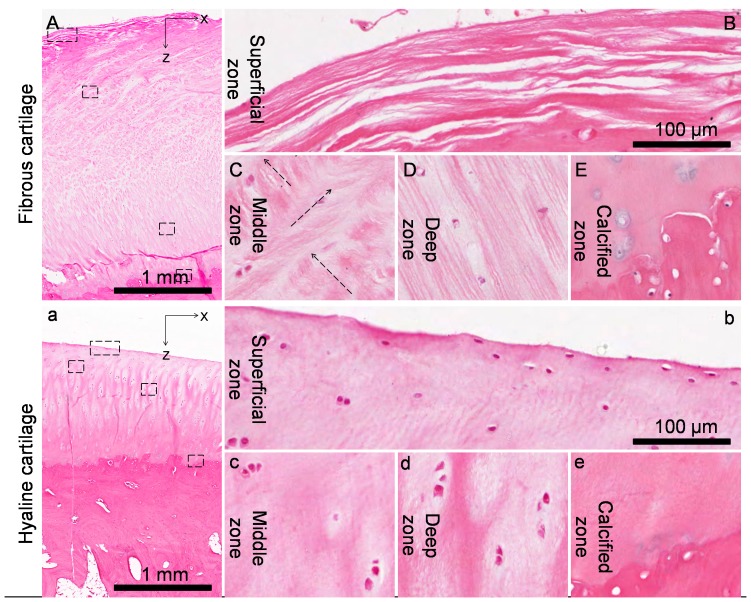
Typical histomorphology of the kangaroo tibial fibrous cartilage and hyaline cartilage. Loose collagen bundles with few chondrocytes were found parallel to the articular surface in the superficial zone (B). Oblique collagen fibres (arrows) were found in the middle zone (C). Long oblique collagen fibres were found in the deep zone (D). No obvious collagen orientation was found in the calcified zone (E). A relatively higher chondrocyte density was found in zones of hyaline cartilage (b–e) but the orientation of collagen fibres was not obvious except in the deep zone where longitudinal collagen fibres were found (d).

The full thickness histological section stained with H&E revealed that the fibrous cartilage was about three times thicker than the peripheral hyaline cartilage (Fig. 2A), and that the chondrocytes were more sparsely distributed in fibrous cartilage than in hyaline cartilage (Fig. 3). Four zones (superficial, middle, deep and calcified) were identified according to the chondrocyte arrangement, and microstructural variations were observed between the fibrous cartilage and hyaline cartilage (Fig. 2A, Fig. 3). In the fibrous cartilage, visible horizontal collagen bundles were loosely arranged in the superficial zone (Fig. 3B); more than one major direction of collagen bundles was found in the middle zone (Fig. 3C); and oblique collagen bundles were found in the deep zone (Fig. 3D). However, the collagen arrangements in the superficial, middle and deep zones of hyaline cartilage were more homogenous and no visible fibres could be identified under the same magnification (Fig. 3b, c & d). No notable difference was observed in the calcified zone between the fibrous cartilage and hyaline cartilage (Fig. 3E & e).

**Figure 4:**
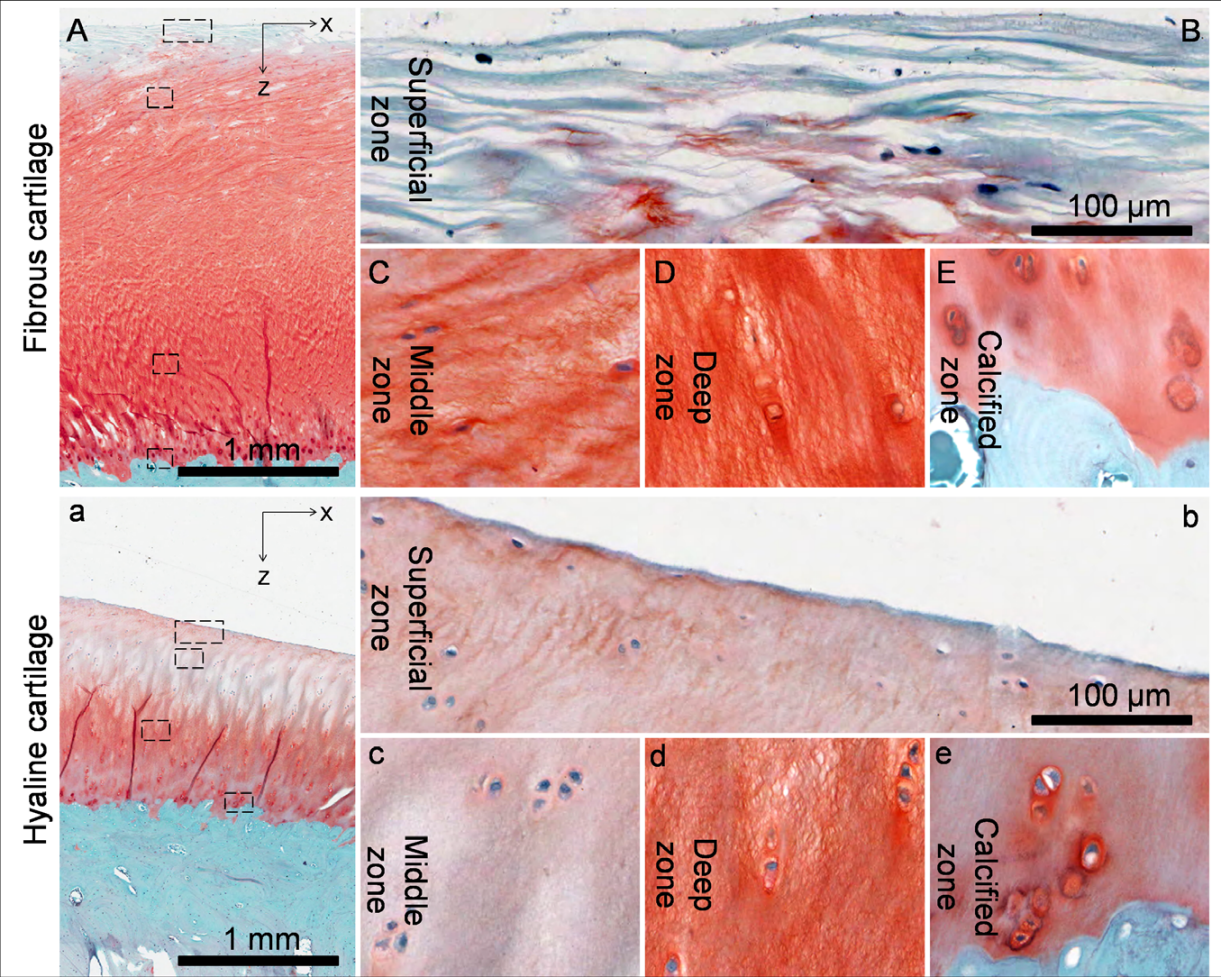
PG distribution of the kangaroo tibial fibrous cartilage and hyaline cartilage indicated by safranin O – fast green staining. In both fibrous cartilage and hyaline cartilage, PG concentration increased from little in superficial zone to a maximum in the deep zone and then decreased in the calcified zone. However, compared to the hyaline cartilage, the fibrous cartilage showed higher PG concentrations in all zones except in the superficial zone.

For both fibrous cartilage and hyaline cartilage, PG concentration increased from the superficial zone to the deep zone (Fig. 2B, Fig. 4). Compared to hyaline cartilage, the fibrous cartilage had higher PG concentrations in the middle and deep zones (Fig. 2B, Fig. 4C & D, c & d). However, the superficial zone of the fibrous cartilage, with little Safranin O staining, had less PG than the superficial zone of hyaline cartilage (Fig. 2B, Fig. 4A & B, a & b).

### Three Dimensional Architecture of Collagen Matrix

**Figure 5:**
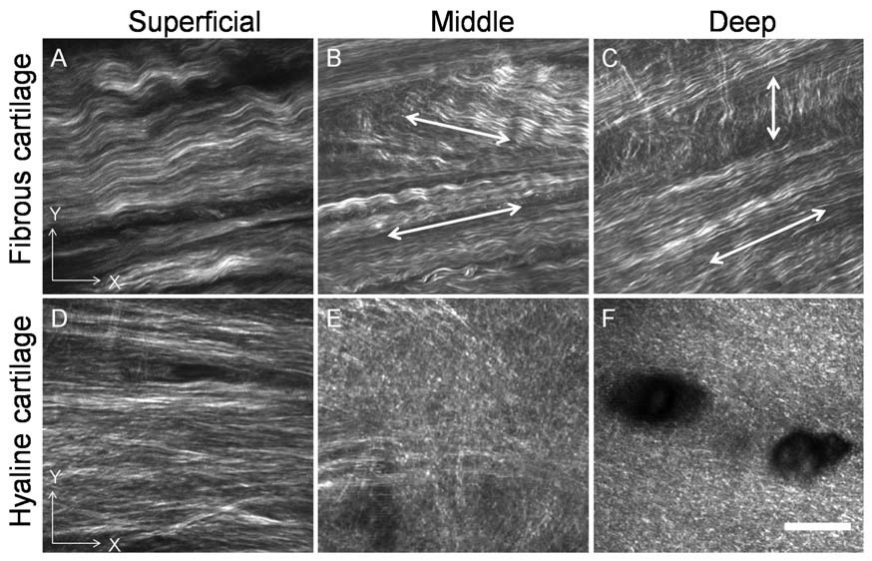
Transverse view of the organization of collagen in kangaroo tibial fibrous and hyaline cartilage. Collagen bundles in the fibrous cartilage were mainly in a wave form and horizontal collagen was found not only in the superficial zone (A) but also in the middle and deep zones (B and C). In the middle and deep zones of the fibrous cartilage the collagen bundles were interwoven in different directions (arrows in B and C). In the hyaline cartilage, horizontal collagen was mainly found in the superficial zone (D). A mix of horizontal collagen and longitudinal collagen was found in the middle zone (E) but no horizontal collagen was found in the deep zone of the hyaline cartilage (F). Scale bar = 20 µm.

**Figure 6:**
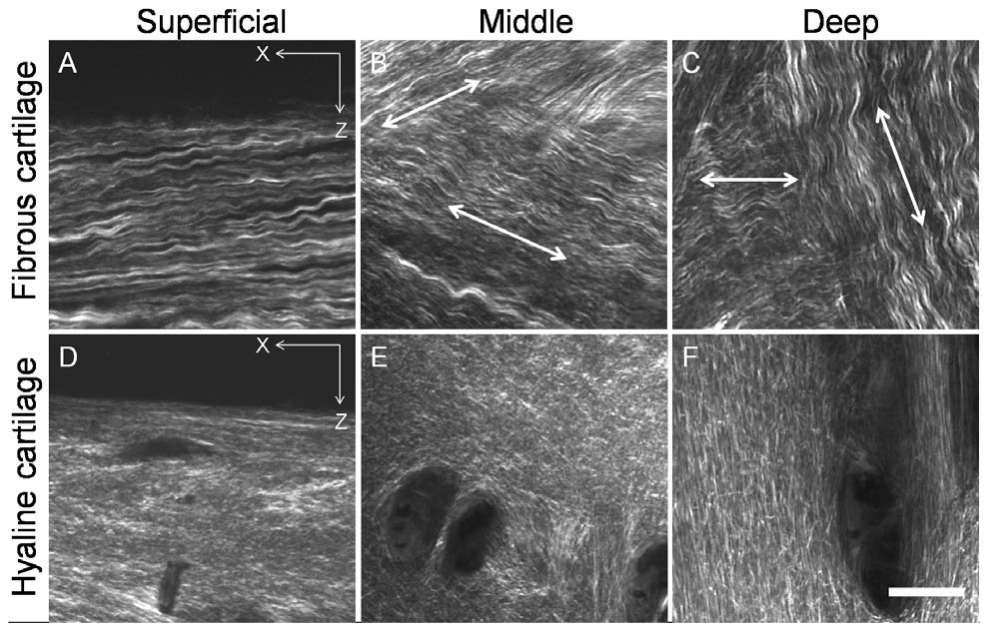
Longitudinal view of the organization of collagen in kangaroo tibial fibrous and hyaline cartilage. In the fibrous cartilage, horizontal collagen was found in the superficial zone (A); a mix of oblique collagen in the longitudinal direction was found in the middle zone (arrows, B); and both horizontal and longitudinal collagen was found in the deep zone (arrows, C). In the hyaline cartilage, horizontal collagen was found in the superficial zone (D); a mix of in different directions was found in the middle zone (B); and longitudinal collagen was found in the deep zone (F). Scale bar = 20 µm.

Figures 5 and 6 show the morphology and organisation of the collagen fibres of the tibial fibrous and hyaline cartilage of the transverse and longitudinal planes. Large collagen bundles in a wave form were predominant in fibrous cartilage (Fig. 5A, B & C, Fig. 6 A, B & C), while collagen fibres in hyaline cartilage were mostly straight (Fig. 5D, E & F, Fig. 6D, E & F). In the fibrous cartilage, collagen bundles in the superficial zone were parallel to the articular surface and oriented from the intercondylar eminence to the peripheral portion (Fig. 5A, Fig. 6A). In the middle zone collagen parallel to the articular surface, seen as interwoven bundles from the transverse view (Fig. 5B), and collagen oblique to the articular surface, seen as interwoven bundles from the longitudinal view (Fig. 6B), were observed to form a complex collagen meshwork. In the deep zone a three dimensional architecture of collagen was formed by both horizontal bundles in the transverse plane (Fig. 5C) and oblique or perpendicular bundles in the longitudinal plane (Fig. 6C).

In contrast, the straight collagen fibres in the hyaline cartilage showed typical Benninghoff structure: parallel to the articular surface in the superficial zone (Fig. 5D, Fig. 6D); randomly organized without a predominant direction in the middle zone (Fig. 5E, Fig. 6E); and perpendicular to the articular surface in the deep zone (Fig. 5F, Fig. 6F).

### The Relationship between Elastin Fibres and Chondrocytes in the Fibrous Cartilage

**Figure 7:**
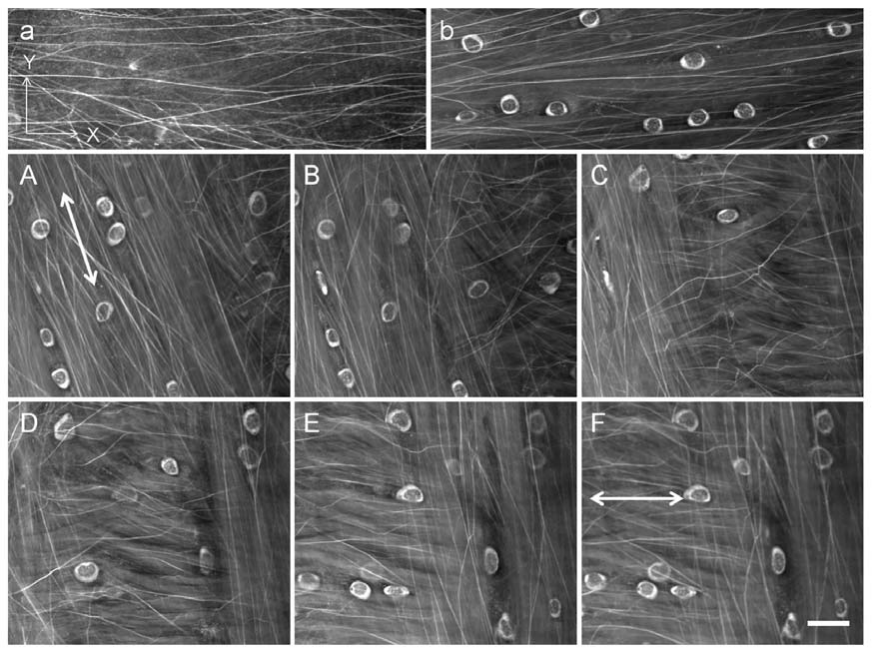
Location of elastin and elastin fibres and the relationship with chondrocytes shown by consecutive images at a step size of 1 µm. Elastin fibre and fine elastin were located in the ECM and around chondrocytes, repectively (a, b). Elastin fibres were aligned parallel to the adjacent chondrocytes (A–F) and changed their orientation with the chondrocytes as depth increased (arrows in A–F). Images were obtained from a transverse view. Scale bar = 20 µm.

From a transverse view, visible elastin fibres in the extracellular matrix (Fig. 7a & b) and fine elastin around the chondrocytes (Fig. 7b) were observed in the fibrous cartilage. These elastin fibres were generally oriented from the intercondylar eminence to the peripheral area of the tibial plateau, but branches and cross-linking were also observed. Consecutive (1 µm step) images showed the orientation of the elastin fibres to match that of chondrocytes interspersed in the elastin fibres (Fig. 7A–F). This relationship remained even through changes in the elastin fibre orientation (arrows, Fig. 6A–F).

### Three Dimensional Architecture of Elastin Fibres

**Figure 8:**
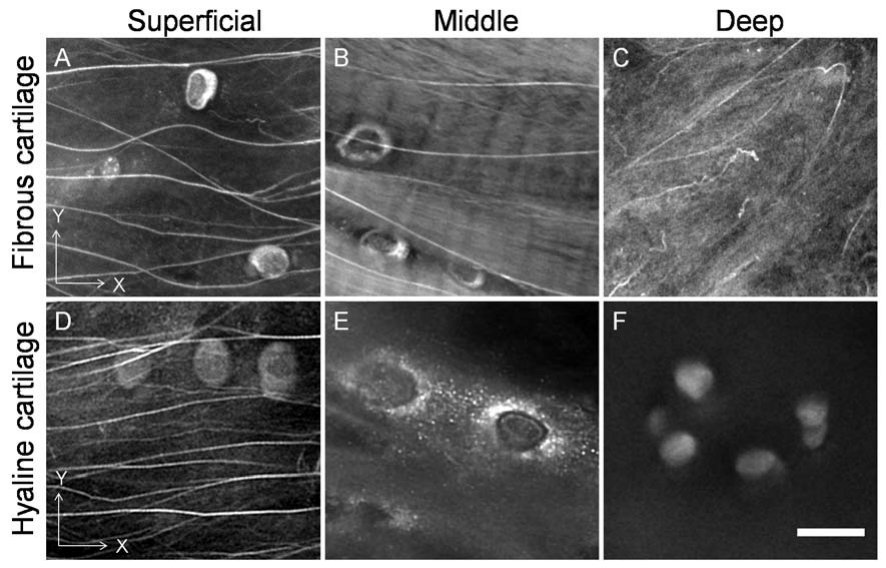
Transverse view of the organisation of elastin fibres in the tibial fibrous and hyaline cartilage. Horizontal elastin fibres were found throughout the tissue depth of the fibrous cartilage, including the superficial (A), middle (B) and deep (C) zones. In the hyaline cartilage, elastin fibres were parallel to the articular surface and only found in the superficial zone (D). There were no resolvable elastin fibres in the ECM but fine elastin around chondrocytes was found in the middle (E) and deep (F) zones. Scale bar = 20 µm.

**Figure 9:**
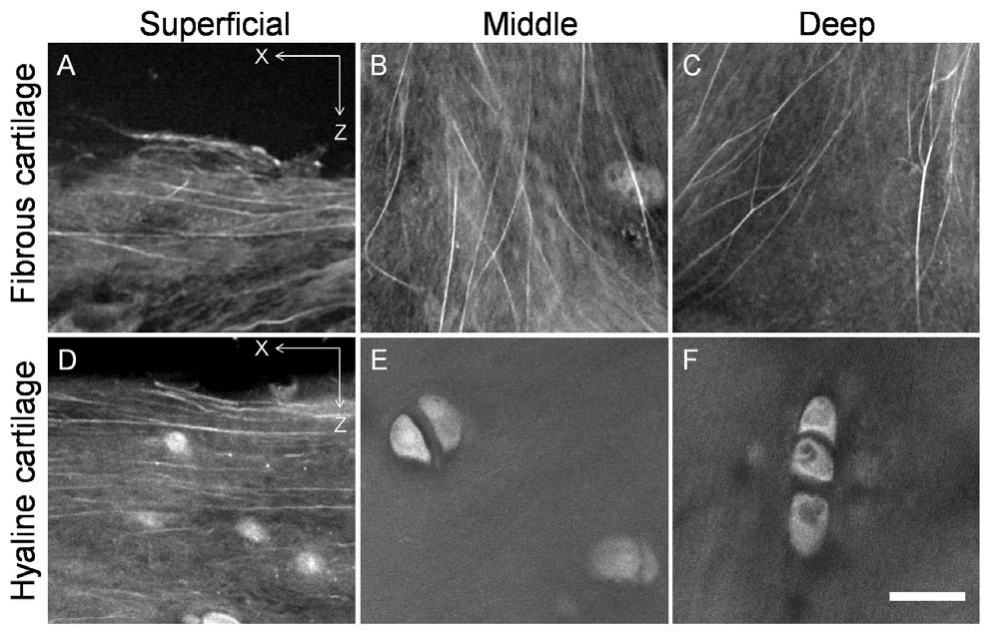
Longitudinal view of the organisation of elastin fibres in the tibial fibrous and hyaline cartilage. In the fibrous cartilage, horizontal elastin fibres were found in the superficial zone (A); longitudinal elastin fibres with branches were found in the middle (B) and deep (C) zones. In the hyaline cartilage, elastin fibres were parallel to the articular surface and only found in the superficial zone (D). There were no resolvable elastin fibres in the ECM but fine elastin around chondrocytes was found in the middle (E) and deep (F) zones. Scale bar = 20 µm.

In fibrous cartilage, the elastin fibres formed a similar three dimensional architecture to the collagen bundles but with a lower density. The fibres were parallel to the articular surface in the superficial zone (Fig. 8A, Fig. 9A). In the middle and deep zones of the fibrous cartilage, both horizontal and oblique to longitudinal elastin fibres were found (Fig. 8B & and C, Fig. 9B and C). These fibres were in one or two major orientations and interconnected by branches (Fig. 8A, B & C, Fig. 9A, B & C), thus forming a three dimensional elastic scaffold in the fibrous cartilage. In hyaline cartilage, however, the elastin fibres were confined to the superficial zone (Fig. 8D, Fig. 9D), and only fine elastin was found around the chondroctyes in the middle and deep zones (Fig. 8E & F, Fig. 9E & F).

## Discussion

The current study examined in detail the microstructure, composition and mechanical properties of the central fibrous cartilage and peripheral hyaline cartilage of the medial tibia of kangaroo.

This study found that the fibrous cartilage articulating directly with the distal femoral condyle was thicker than the periphery hyaline cartilage covered by meniscus. This suggests that the unique tibia anatomy may be attributed largely to the kangaroo knee joints to afford unusual locomotion of the animals generating high frequently dynamic shear and compressive stresses. This is consistent with the previous findings by other scholars that the existence of the fibrous cartilage pad on the kangaroo tibial plateau was important for kangaroo knees to adapt to the high compression and shear forces generated during high speed hopping [2].

PG concentration in hyaline cartilage determines the compressive resistance of the tissue [13]–[15]. Thus, a higher PG concentration in the fibrous cartilage pad than the periphery hyaline cartilage found in this study suggested the fibrous cartilage pad has a high compressive modulus than the periphery hyaline cartilage during joint articulation. However, the pin creep indentation tests showed that the fibrous cartilage had a lower compressive modulus than the surrounding hyaline cartilage. The may be several reasons for this discrepancy. The low stress used in these tests may be more reflective of the compressive modulus in the toe region of other published creep data and thus may not represent the compressive loading conditions during real-life joint articulation. It is also possible that the small indenter may have an effect as pilot studies showed complete collapse of the fibrous cartilage at relatively low loads, indicating that perhaps the small diameter indenter may have penetrated into between the fibres of the fibrous cartilage. Thus, a larger indenter might suit the relatively softer fibrous cartilage. It is also possible that while the PG content predicts the compressive modulus in hyaline cartilage this relationship may not hold in the differently structured fibrous cartilage.

The fibrous cartilage pad contained a distinctive 3D collagen meshwork compared to the peripheral hyaline cartilage. The collagen in articular cartilage is conventionally believed to act primarily in tension, and constrain the swelling pressure of PG to provide the load capacity of AC [16]. The heterogeneous architecture of the collagen fibres is particularly important to the compressive, shear and wearing strength of AC. The distinctive collagen matrix found in the fibrous cartilage is consistent with the unique requirement of the mechanical properties of the fibrous cartilage pad for withstanding for the periodic loading, compressive and shear stresses generated from kangaroos’ normal activities. The superficial zone contained waveform collagen fibres oriented predominantly in a spatial direction oblique to the articular surface (Figure.6 A) to maximise the tensile strength of the surface. In the middle zone, waveform collagen fibres formed as bunches aligned in two distinctive orientations were interwoven into a three dimensional collagen meshwork (Figure 6. B), which would offer sufficient support for dynamic bouncing and shear stresses. In the deep zone, waveform collagen fibres formed into collagen bunches oriented in two directions parallel and perpendicular to the articular surface (Figure.6 C). This structure is thought to offer excellent support for bouncing and compressive stresses. The waveform of the collagen fibre in tendons and ligaments has been suggested to permit the tissue to absorb tensile deformations efficiently [17]. Therefore, the waveform collagen fibres found in the fibrous cartilage may play a similar role of the collagen fibres in tendons and ligaments.

The function of elastin fibres found in skin, lungs, arteries, veins, elastic cartilage, ligament [18]–[20] and the surface of articular cartilage [21]–[23] have been suggested to endow the tissues with elastic recoil and resilience for efficient recovery from mechanical deformation [18]–[24]. The elastin in the lumbar annulus fibrosus enhances the mechanical integrity of the tissue [25]. The current study confirmed a similar elastic network confined within the surface of the kangaroo hyaline cartilage. In comparison, abundant elastin fibres were found from the surface to deep zone of the fibrous cartilage forming a complex elastic framework throughout the fibrous cartilage. Thus, this study suggests that the fibrous cartilage pad contained an elastic network parallel to the collagenous scaffold, which may biomechanically function to allow the tissue to deform and recover efficiently during kangaroos’ locomotion.

The biological roles of elastin vary between tissues: controlling proliferation of vascular smooth muscle and stabilizing arterial structure [19]; mediating disease states of Supravalvular aortic stenosis [26], [27] and Williams syndrome [28]. Based on the biological importance of elastin in many tissues [19], [24]–[28] and increasing evidence of the presence of elastin in hyaline cartilage [21]–[23], it is necessary to investigate in details the role of elastin in both the fibrous cartilage and hyaline cartilage and its relationship with the local mechanical environments. Also, a previous study has shown *in vitro* that cell orientation determines the alignment of the cell-produced collagenous matrix [29]. Elastin fibres were found to be parallel to the alignment of chondrocytes in this study, altering direction with the chondrocytes as depth increased (Fig. 7A–F). A close relationship between the chondrocytes and elastin fibres is therefore expected although we did not investigate whether chondrocytes could also determine the orientation of elastin fibres. The detailed interaction between elastin fibres and chondrocytes is not currently resolvable due to limitations of microscopy techniques, but the findings from this preliminary study merit further investigation of the association of elastin fibres with chondrocytes and other ECM components.

In conclusion, tibial fibrous cartilage differs from the peripheral hyaline cartilage with regard to the organization of chondrocytes and major ECM components. The unique architecture of collagen and elastin fibres could provide the fibrous cartilage with improved compression absorption, extensibility, elasticity and resilience. These characteristics of fibrous cartilage may render the kangaroo tibial plateau to withstand the great tensile, shear and compressive stresses generated in the knee joints during kangaroo hopping. Further investigations would be valuable to address the uniqueness of kangaroo tibia as compared to other species and the specific roles of elastin fibres in the physiology and pathology of articular cartilage.
